# Kinetic Study on the Base-Catalyzed Imine-Enamine Tautomerism of a Chiral Biologically Active Isoxazoline Derivative by HPLC on Amylose Tris(3,5-dimethylphenylcarbamate) Chiral Stationary Phase

**DOI:** 10.3390/molecules28186518

**Published:** 2023-09-08

**Authors:** Daniele Sadutto, Paolo Guglielmi, Simone Carradori, Daniela Secci, Roberto Cirilli

**Affiliations:** 1 Centre for the Control and Evaluation of Medicines, Chemical Medicines Unit, Istituto Superiore di Sanità, Viale Regina Elena 299, 00161 Rome, Italy; daniele.sadutto@iss.it; 2 Department of Drug Chemistry and Technologies, Sapienza University of Rome, 00185 Rome, Italy; paolo.guglielmi@uniroma1.it (P.G.); daniela.secci@uniroma1.it (D.S.); 3 Department of Pharmacy, “G. d’Annunzio” University of Chieti-Pescara, 66100 Chieti, Italy; simone.carradori@unich.it

**Keywords:** isoxazoline, chiral stationary phase, stereointegrity, imine, enamine, base-catalyzed tautomerization, Chiralpak IA

## Abstract

Isoxazoline is a nitrogen- and oxygen-containing five-membered heterocyclic scaffold with diverse biological profiles such as antimicrobial, fungicidal, anticancer, antiviral, analgesic and anti-inflammatory activity. Accordingly, the use of this peculiar structural framework in drug discovery is a successful strategy for the development of new drug candidates. Here, a chiral saccharin/isoxazoline hybrid was considered to investigate the tendency of the imine moiety of the heterocyclic ring to tautomerize to the enamine form in the presence of a basic catalyst. The pseudo-first-order rate constants for the base-catalyzed tautomerization process were measured in different solvents and at different temperatures by off-column kinetic experiments based on the amylose (3,5-dimethylphenylcarbamate)-type chiral stationary phase. The kinetic results obtained in this study may be a useful aid in the perspective of designing experimental conditions to control the stereointegrity of these types of pharmacologically active compounds and drive their synthesis toward the preferred, imine or enamine, tautomer.

## 1. Introduction

High-performance liquid chromatography (HPLC) on chiral stationary phase (CSP) is an effective and direct enantioseparation technique. It is widely used on a laboratory and industrial scale to determine the enantiomeric excess (ee) of natural and synthetic chiral compounds, including enantiopure drugs, and to isolate enantiopure forms on a semi-preparative and preparative scale [1].

The general mechanism of chiral discrimination is based on the transient formation of diastereomeric adducts between each enantiomer of the chiral sample to be chromatographed (the selectand) and the chiral structure bound to the chromatographic support (the selector).

At the microscopic level, enantioseparation can be achieved when a pair of resolvable enantiomers exploit enantiospecific differences in interaction with the active sites of a chiral selector. The thermodynamic driving force that enables enantioseparation is the energy difference between two transient diastereomeric enantiomers/CSP complexes (ΔΔG°). Typically, the range of interest for HPLC applications requires the performance of discriminations characterized by ΔΔG° values greater than 0.11 kcal mol^−1^, an amount of energy that, according to the following equation, ΔΔG° = −RT × ln(α), corresponds to enantioseparation factor (α) values greater than 1.2. The intermolecular interactions controlling the energy of the diastereomeric complexes are both attractive and repulsive, e.g., H-bonds, dipole-dipole, van der Waals, π-π, ionic and steric interactions [2]. However, it must be emphasized that even if the chiral recognition process on a given CSP is thermodynamically favored, it may be hampered or precluded by the establishment of competitive on-column secondary dynamic processes involving the enantiomers and occurring on the time scale necessary to achieve the desired resolution [3,4,5,6]. The existence of such equilibria leads to the production of de novo species and therefore to potential peak splitting, which, in turn, if the competitive process proceeds at a rate similar to that of the separation process, will also be characterized by the presence of plateau zones between interconverting species. Such aberrations of the chromatographic profile are considered an obstacle to separation, but in other types of studies, such as those performed by dynamic HPLC, they may be indicative of the stereochemical lability of the analyte and may be used to determine the energy barrier of the competitive process [7,8,9].

The enantiomerization of stereolable enantiomers [3,7,8,9] and the tautomerization of keto-enols [10,11] and imine-enamines [12] are well-established dynamic on-column processes, which, in the first, case refer to the inversion of the absolute configuration of a chiral analyte and, in the second case, to an equilibrium between two tautomeric species, both occurring on the separation time scale.

Thus, as a function of the chosen experimental HPLC conditions, the primary distribution equilibria between CSP and the mobile phase and the secondary equilibria can compete with each other and, depending on which of them is the dominant process, allow or suppress the observed separation. The instability of enantiomers or isomers is a topic of interest for academia and pharmaceutical companies when the species affected by the phenomenon of interconversion is a biologically active compound. In this regard, since 1993, drug regulatory authorities have considered it mandatory to provide comprehensive information on the stereochemical stability of those drugs that are administered as individual enantiomers or isomers [13].

It is therefore necessary to study the kinetics of configurational interconversion and the factors that influence the stability of the individual stereoisomers of biologically active molecules.

**Figure 1:**
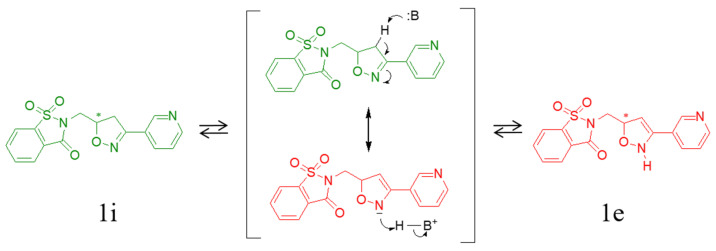
Base-catalyzed imine(**1i**)-enamine(**1e**) tautomerism mechanism of **1**.

From this perspective, the aim of this work is to study the configurational stability of compound **1** (Figure 1), chosen as a representative of a class of saccharin-isoxazoline derivatives. The choice of this chemical class was based on two aspects. First, the isoxazoline derivatives are characterized by important pharmacological activities such as antimicrobial, anti-inflammatory, antiviral, analgesic, antithrombotic and antitumor activities; therefore, they could be chosen as an important basic scaffold in the development of new pharmaceutical compounds [14]. Second, isoxazolines are widely used as advanced intermediates in the synthetic process of bioactive compounds due to their high chemical stability and hydrogen bonding ability. In particular, compound **1** (Figure 1) has shown an interesting inhibitory and selective activity toward enzymatic isoforms CAIX and CAXII of carbonic anhydrase (CA). Due to this peculiar biological activity, some analogs of **1** are candidates as antitumor agents [15]. 

As shown in Figure 1, two critical fragments of the isoxazoline cycle are potentially of interest for stereoisomerization, namely the C_5_ stereogenic center and the unconjugated imine double bond. 

Enantiomers of saccharine-isoxazoline derivatives have shown a high enough stereochemical stability to allow their chromatographic separation at room temperature [15]. On the other hand, the tendency of isomerization of the imine moiety into the enamine form, which is an important structural element for biological activity, remains to be evaluated.

In this work, the kinetic parameters of the base-catalyzed imine-enamine interconversion of **1** (Figure 1) were determined by a classical batchwise operational approach combined with enantio- and tautomer-selective HPLC. The amylose-based Chiralpak IA was chosen as the chiral stationary phase. Kinetic studies were carried out in some organic protic and aprotic solvents containing a basic catalyst and at different temperatures.

## 2. Results

### 2.1. Base-Catalyzed Imino-Enamine Tautomerism Studied by HPLC on Chiral Stationary Phase

The first step of our study was to evaluate the chiral recognition ability of the amylose (3,5-dimethylphenylcarbamate)-based Chiralpak IA column under polar organic conditions. The amylose derivative is considered one of the most effective chiral selectors for achieving chiral resolution [16,17,18]. 

**Figure 2:**
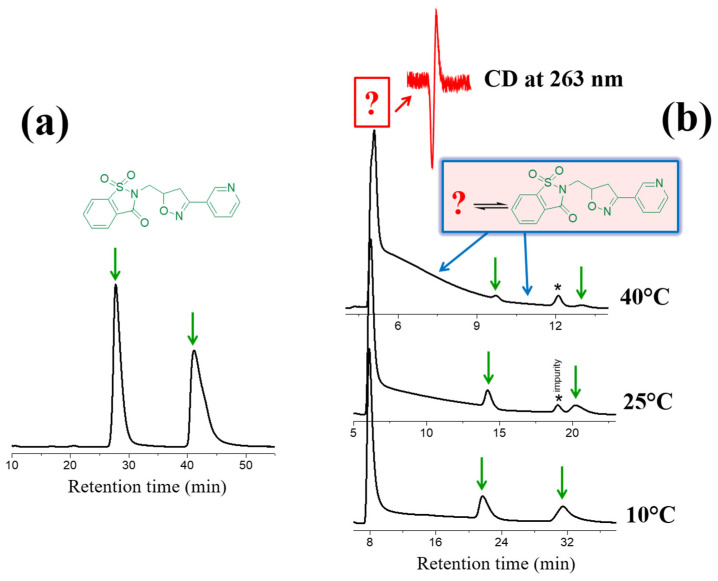
HPLC of **1** using pure methanol (**a**) and methanol:DEA 100:0.6 (*v*/*v*) as mobile phases (**b**). Chromatographic conditions: column, Chiralpak IA (250 mm × 4.6 mm, 5 μm); column temperature: 25 °C (**a**) and 10, 25 and 40 °C (**b**); flow rate: 0.8 mL/min; detection, UV/CD at 263 nm.

A baseline separation of the enantiomers of **1** was achieved using pure methanol as a mobile phase. As can be seen in Figure 2a, the profile of the chromatogram did not show the typical deformation resulting from on-column secondary configurational interconversion processes [3,4,5,7,8,9]. 

This indicates that the compound was configurationally stable under the conditions and analysis times employed for chromatographic separation. Conversely, a dramatic change in the elution profile of the isoxazoline derivative was observed when a basic species, such as diethylamine (DEA), was added to the alcoholic mobile phase (Figure 2b).

It is possible to observe how, at a temperature of 10 °C, using the mixture methanol:DEA 100:0.6 (*v*/*v*) as a mobile phase, a new chemical species was clearly visible, eluted before the imine enantiomeric peaks (**1i**) and connected to them by a plateau region. The height of the plateau gradually increased by gradually increasing the temperature of the chromatography column up to 40 °C. The generated plateau zone was formed by the chemical species that underwent the secondary process of interconversion at least once during the chromatographic separation; the initial and terminal peaks instead corresponded to the molecules that maintain their configuration throughout the resolution process.

Looking at the chromatogram detected by monitoring the sign of CD at 263 nm during chromatography (Figure 2b), it is interesting to note that, at 40 °C, the peak corresponding to the novo chemical species split into a pair of bisegnated peaks. This indicates that the species resulting from the dynamic interconversion process were chiral.

From this evidence, it is possible to hypothesize that the dynamic process is a base-catalyzed imine–enamine (I⇆E) tautomerization. The base-catalyzed imine-enamine tautomerization involves two steps (Figure 1): the removal of a proton from the base to form a resonance-stabilized carbanion and the subsequent transfer of the proton, which can bind to the starting carbon or nitrogen.

**Figure 3:**
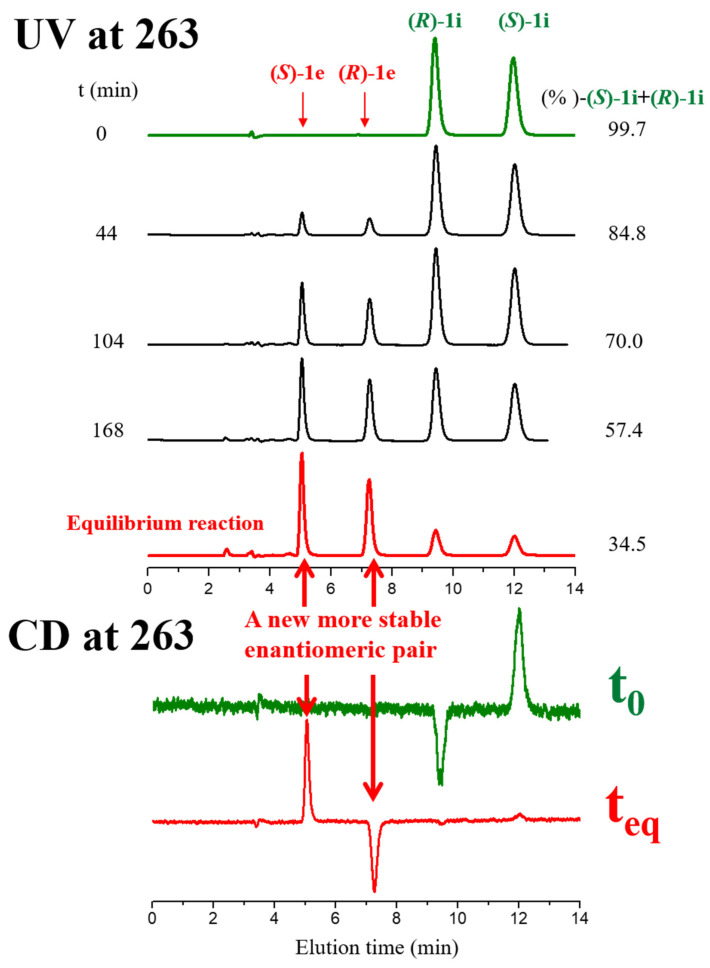
Base-catalyzed off-column tautomerization of *rac*-1 monitored by HPLC. Solvent reaction: ethanol-TEA 92:8 (*v*/*v*); temperature reaction: 60 °C. Chromatographic conditions: column, Chiralpak IA (250 mm × 4.6 mm, 5 μm); mobile phase, ethanol-ethyl acetate-DEA 100:20:0.1 (*v*/*v*/*v*); column temperature: 25 °C; flow rate: 0.8 mL/min; detection, UV/CD at 263 nm.

To verify this hypothesis and to quantitatively evaluate the configurational stability of the isooxazoline compound, a series of off-column kinetic experiments were performed. Samples of **1** in enantiomeric or racemic forms were incubated at the desired temperature in a suitable reaction solvent containing triethylamine (TEA) as a basic catalyst. At recorded time intervals, the chiral samples were analyzed by HPLC on the Chiralpak IA CSP in stereoselective conditions that quenched the interconversion (mobile phase: ethanol-ethyl acetate-DEA 100:20:0.1 (*v*/*v*/*v*); column temperature: 25 °C; flow rate: 0.8 mL/min). Under these conditions, the competitive on-column base-catalyzed tautomerism did not occur on the time scale of the chromatographic separation, as indicated by the absence of plateau-like regions between two well-resolved peak pairs (Figure 3). 

As shown in Figure 3, the areas of the third and fourth eluted peaks, corresponding to the imine form of **1** (**1i**), decreased; at the same time, two enantiomeric peaks, corresponding to the enamine form (**1e**), appeared, and their areas progressively increased over the isomerization reaction time. Thus, the isomeric ratio (calculated at the isosbestic point; see Figure S1 in the Supplementary Materials) changed progressively with time and, at equilibrium, the tautomeric enamine form was the most stable (64.5%). The evaluation of the signs and areas of the peaks obtained by online circular dichroism (CD) measurements was the empirical tool to track the isomerization process. The peaks in enantiomeric relationships were easily identifiable because they had the same area but opposite signs. Therefore, (i) each chromatogram showed two pairs of peaks corresponding to four stereoisomers, and (ii) each pair of peaks with the same area and opposite CD sign was in an enantiomeric relationship. 

**Figure 4:**
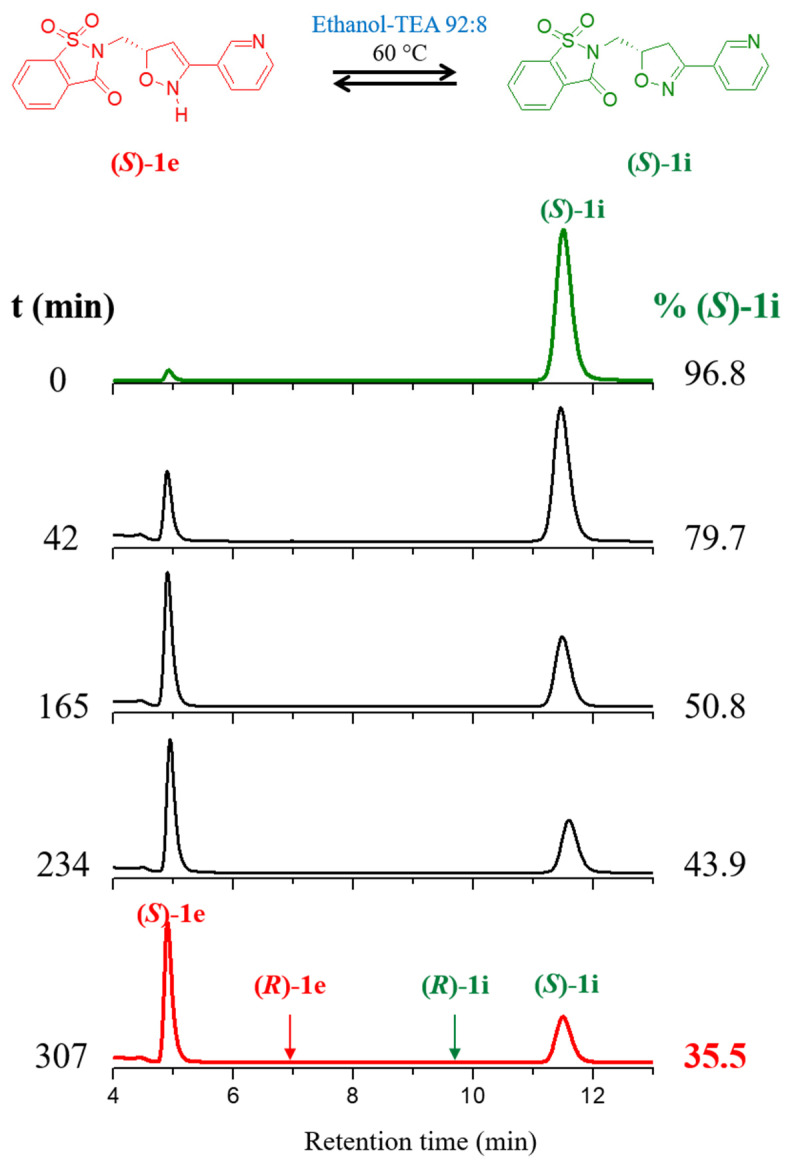
Base-catalyzed off-column tautomerization of (*S*)-**1i** monitored by HPLC. Solvent reaction: ethanol-TEA 92:8 (*v*/*v*); temperature reaction: 60 °C. Chromatographic conditions: column, Chiralpak IA (250 mm × 4.6 mm, 5 μm); mobile phase, ethanol-ethyl acetate-DEA 100:20:0.1 (*v*/*v*/*v*); column temperature: 25 °C; flow rate: 0.8 mL/min; detection, UV/CD at 263 nm.

By repeating the same kinetic experiment but replacing the racemic mixture with the enantiopure (*S*)-**3i**, it was possible to demonstrate that the conversion of the imine into the enamine form proceeded without concomitant racemization. In fact, the absence in the chromatographic patterns of the peaks corresponding to the isomers (*R*)-**1e** and (*R*)-**1i** excluded the simultaneous stereochemical inversion at the stereocenter C_5_ of the heterocyclic moiety (Figure 4).

Since the isomerization reaction did not involve cleavage of any of the bonds of the stereogenic center, (*S*)-**1i**, corresponding to the fourth peak, produced the first eluting isomer (*S*)-**1e**, and consequently the second and third eluting isomers shared the *R* configuration. The resulting isomeric elution order on the Chiralpak IA CSP was as follows (*S*)-**1e** < (*R*)-**1e** < (*R*)-**1i** < (*S*)-**1i**. The peaks pertinent to the (*S*)-**1e**/(*S*)-**1i** and (*R*)-**1e**/(*R*)-**1i** couples recorded by online CD measurements at 263 nm had positive and negative signs, respectively. 

**Figure 5:**
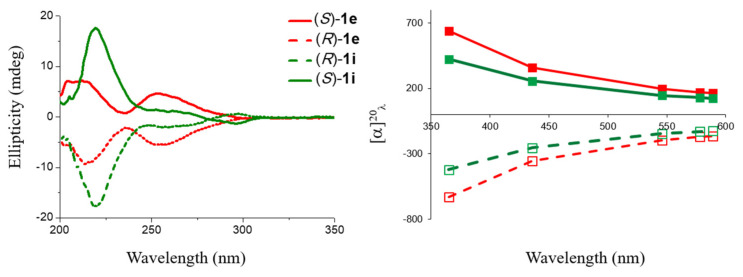
CD spectra and ORD curves of isomers (*S*)-**1e**, (*R*)-**1e**, (*R*)-**1i**, (*S*)-**1i** recorded in ethanol solution.

Figure 5 shows the CD spectra and the optical rotatory dispersion (ORD) curves of four isomers of **1** recorded in ethanol. As expected, the trend of the chiroptical properties as a function of the wavelength for each couple of enantiomers was perfectly specular. It is worth noting that imine/enamine isomers with the same absolute configuration shared the sign of the specific rotation. 

### 2.2. Kinetics of Base-Catalyzed Imino-Enamine Isomerization 

The kinetics of the base-catalyzed imine→enamine (*I*→*E*) and enamine form→imine form (*E*→*I*) isomerization reactions of compound **1** were determined in different solvents (methanol, ethanol, 1-propanol, ethyl acetate, acetonitrile) containing variable concentrations of TEA, selected as a basic catalyst. The solutions were incubated at different temperatures when methanol and ethanol were used.

The isomerization reactions were monitored until the thermodynamic equilibrium between the imine and enamine forms was reached. 

**Figure 6:**
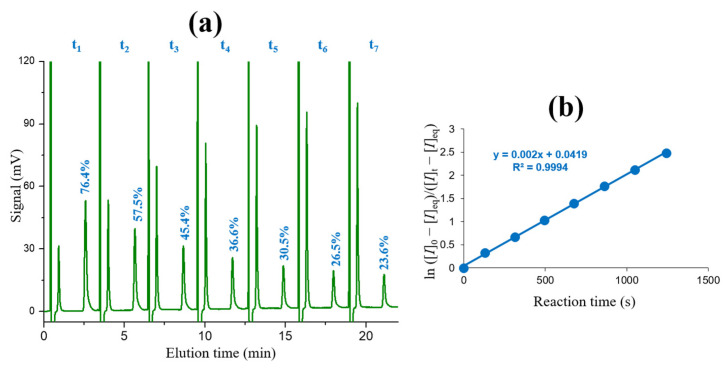
(**a**) Base-catalyzed off-column tautomerization of (*S*)-**1i** monitored by HPLC. Solvent reaction: methanol-TEA 100:0.4 (*v*/*v*); temperature reaction: 30 °C. Chromatographic conditions: column, Chiralpak IA-3 (100 mm × 4.6 mm, 3 μm); mobile phase, n-hexane-ethanol-ethyl acetate-DEA 50:50:20:0.1 (*v*/*v*/*v*/*v*); column temperature: 25 °C; flow rate: 3.5 mL/min; detection, UV/CD at 263 nm. (**b**) time-dependent decay of the (*S*)-**1i** form.

The equilibrium constants (*K_eq_*), related to the process *I*⇆*E*, were calculated according to the following equation:

*K_eq_* = *k_I_*_→*E*_/*k_E_*_→*I*_ = [*E*]_eq_/[*I*]_eq_ (1)

where [*I*]_eq_ and [*E*]_eq_ are the concentrations of imine (*I*) and enamine (*E*) forms at equilibrium, respectively, while *k_I_*_→*E*_ and *k_E_*_→*I*_ are the pseudo-first-order kinetic constants for the *I*→*E* and *E*→*I* reactions, respectively. The sums of the rate constants for the forward and backward isomerization reactions (*k_sum_*) were calculated according to Equation (2):

ln ([*I*]_0_ − [*I*]_eq_)/([*I*]_t_ − [*I*]_eq_) = − (*k_E_*_→*I*_ + *k_I_*_→*E*_) × *t* = − (*k_sum_*) × *t* (2)

where [*I*]_0_, [*I*]_eq_ and [*I*]_t_ are the concentrations of imine form (I) at time zero, equilibrium and time *t*, respectively. Plots traced according to Equation (2), in which the term ln ([*I*]_0_ − [*I*]_eq_)/([*I*]_t_ − [*I*]_eq_) was plotted against the reaction time (Figure 6b), showed highly linear correlations (R^2^ > 0.990). The kinetic constants *k_E_*_→*I*_ and *k_I_*_→*E*_, obtained by the values of *K_eq_* and *k_sum_*, as well as the corresponding half-lives (*t*_0.5_), are shown in Table 1. 

The use of the 10 cm-length Chiralpak IA-3 column allowed us to set high flow rates (3.5 mL/min) and monitor very fast isomerization reactions. In the case of the base-catalyzed (*S*)-**1i**→(*S*)-**1e** reaction shown in Figure 6a, the half-life was only 7 min. 

By analyzing the experimental kinetic data shown in Table 1, it is possible to identify the main factors influencing the rate of base-catalyzed isomerization. First, the nature of the solvent plays a predominant role in this type of reaction. Proceeding along the methanol/ethanol/1-propanol series of alcoholic solvents, the rate decreased sharply. To understand how strongly the nature of the alcoholic solvent affects the rate of isomerization, it is sufficient to observe the changes in the half-lives. For example, the half-life of the *I*→*E* reaction in the presence of methanol/TEA (0.4%) at 35 °C was only 4 min, while in ethanol/TEA (10%) at 50 °C, it increased to 189 min. The same reaction in 1-propanol/TEA (10%) at a higher temperature than the previous ones (60 °C) was practically blocked since the concentration of the imine form did not change after 24 h.

Contrary to what was observed in ethanol and methanol, in the presence of an aprotic polar solvent such as ethyl acetate or acetonitrile, the imine form is configurationally stable and does not isomerize to the enamine form, even in the presence of high concentrations of TEA (10%) (reaction at 60 °C). 

Temperature also has a marked effect on the rate of isomerization. Pseudo-first order rate constants increased with the increasing temperature in both ethanol and methanol (Table 1).

Furthermore, the concentration of the imine form (less stable in ethanol and methanol than the enamine form) at equilibrium decreased with the decreasing temperature. For example, as shown in Figure S2, the content of **1i** in the isomeric mixture at equilibrium gradually decreased from 23% at 50 °C to 6% at −23 °C. 

In the final step of the work, to assess the configuration stability under physiological conditions, the *I*⇆*E* reaction was monitored in methanol solutions containing increasing percentages (5, 15, 30, 60, 80 and 100%) of 10 mM phosphate buffer at pH 7.4 and temperature of 37 °C.

**Figure 7:**
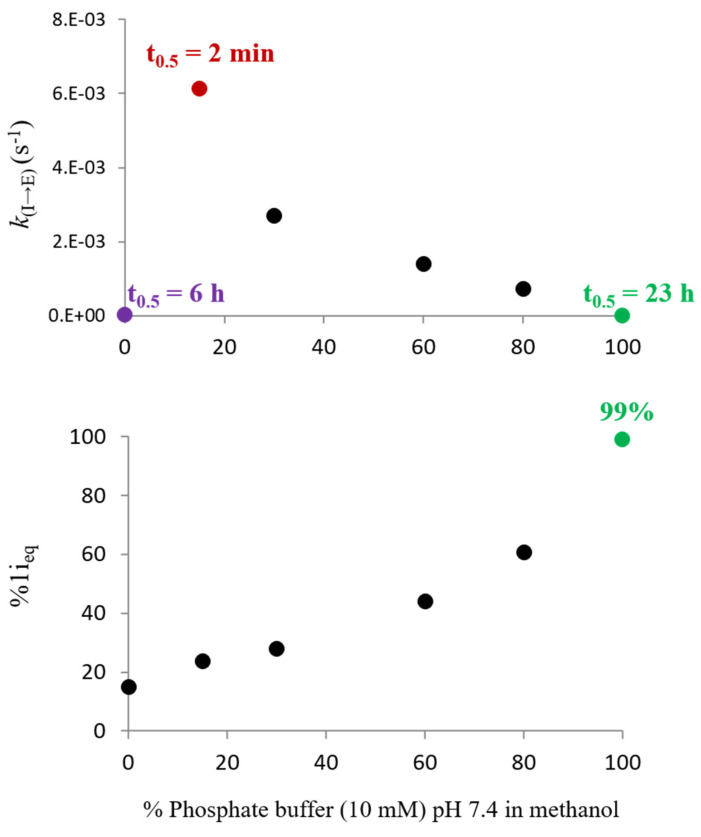
Effect of pH 7.4 phosphate buffer on the rate of the **1i**→**1e** reaction and % **1i**_eq_.

Figure 7 shows the influence of the percentage of buffer on the pseudo-first order constants and the percentage of **1i** at equilibrium. The addition of 15% of 10 mM buffer to the methanolic solvent resulted in a significant increase in the rate of the *I*→*E* reaction; under these conditions, the maximum value of the reaction rate was reached. By increasing the concentration of buffer in methanol, a gradual decrease in the rate of isomerization was observed. In the presence of 100% phosphate buffer, the process was instead very slow, slower than that observed in pure methanol.

It is important to note that, while in the methanolic solution, the most stable form was the enamine (72%) with a half-life of 6 h; under biomimetic conditions, the equilibrium was completely shifted in favor of the imine (99% of the total), and the half-lives of **1i** and **1e** were 23 h and 14 min (Figure 7).

## 3. Materials and Methods

### 3.1. Materials

Methanol, ethanol, 2-propanol and acetonitrile, diethylamine and triethylamine were purchased by Sigma-Aldrich (Milan, Italy). Chiral HPLC separations were performed on the Chiralpak IA (250 mm × 4.6 mm I.D., 5 μm), Chiralpak IA-3 (100 mm × 4.6 mm I.D., 3 μm) and Chiralpak IA (250 mm × 10 mm I.D., 5 μm) columns purchased from Chiral Technologies Europe (Illkirch, France).

### 3.2. Chromatographic Analysis 

The analytical analysis was performed by a high-performance liquid chromatography (HPLC) apparatus consisting of a Perkin-Elmer pump (LC 2000 series) (Norwalk, CT, USA), Rheodyne injector (Cotati, CA, USA), 50 μL sample loop, Jasco CD 2095 Plus UV/CD detector (Tokyo, Japan) and Perkin-Elmer LC 101 HPLC thermostat (Sunnyvale, CA, USA).

For semipreparative separation, the HPLC apparatus was similar to the previous one but with a different sample loop capacity (2000 μL) and detector (UV Waters 484, Waters Corporation, Milford, MA, USA). All data were acquired and processed by ClarityTM software DataApex (Prague, Czech Republic).

### 3.3. Chiroptical Analysis

The CD spectra of the enantiomers of **1i** and **1e** were recorded using a Jasco (Jasco, Tokyo, Japan) J-700 spectropolarimeter. The enantiomers, isolated on a semi-preparative scale, were dissolved in ethanol at a concentration of approximately 0.3 mg/mL and successively transferred to a quartz cell (0.1 cm path length) at 25 °C. The CD spectra were averaged over three instrumental scans, and the intensities are expressed as ellipticity values (mdeg).

The specific optical rotations of the enantiomers of **1i** and **1e** were determined using a Perkin Elmer 241 polarimeter equipped with sodium and mercury lamps. A 1 mL cell with an optical path of 10 cm was used. The system was thermostated at 20 °C using a Neslab RTE 740 cryostat.

### 3.4. Synthesis of Compound ***1i***

Solvents and reagents were employed as provided by the vendors without further purification (Sigma-Aldrich^®^, Milan Italy). Where mixtures of solvents are specified, the stated ratios are volume:volume. ^1^H and ^13^C NMR spectra were recorded at 400 MHz on a Bruker spectrometer using CDCl_3_ as the solvent at room temperature. Chemical shifts are expressed as *δ* units (parts per millions) relative to the solvent signal. ^1^H spectra are reported as follows: δ_H_ (spectrometer frequency, solvent): chemical shift/ppm (multiplicity, J-coupling constant(s), number of protons, assignment). Multiplets are abbreviated as follows: br—broad; s—singlet; d—doublet; t—triplet; q—quartet; m—multiplet. Coupling constants *J* are valued in Hertz (Hz). Purification on column chromatography was carried out using silica gel (high purity grade, pore size 60 Å, 230–400 mesh particle size). All the operations were monitored by TLC performed on 0.2 mm-thick silica gel-aluminium backed plates (60 F_254_, Merck, Lowe, NJ, USA). Visualization was carried out under ultra-violet irradiation (254 nm). Where given, systematic compound names are those generated by ChemBioDraw Ultra 12.0 following IUPAC conventions. 

**Scheme 1:**
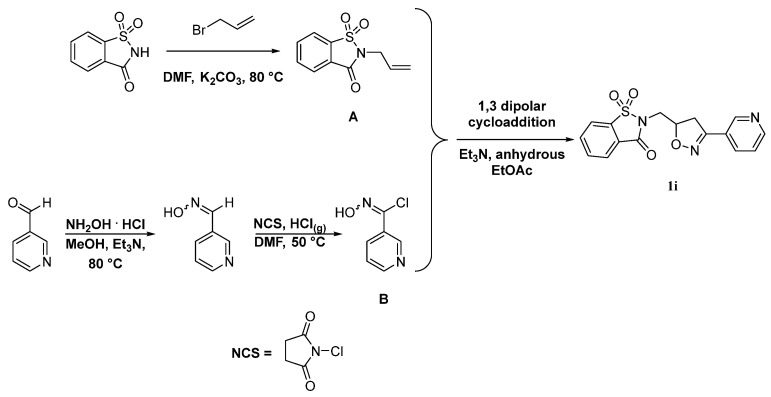
Synthesis of the compound **1i**.

The synthesis and chemical characterization of compound **1i** was accomplished through a multistep approach, taking advantage of our previously reported procedure (Scheme 1) [15]. Briefly, *N*-allyl saccharin (**A**) was obtained through nucleophilic substitution between saccharin and allyl bromide, while the hydroximinoyl acid chloride (*N*-hydroxynicotinimidoyl chloride, **B**) was synthesized in two steps from the corresponding benzaldehyde (Scheme 1). *N*-allyl saccharin (**A**) and the *N*-hydroxynicotinimidoyl chloride (**B**) were the final reagents to perform the 1,3-dipolar cycloaddition, leading to the title compound **1i**.

Compound **1e** was obtained through the base-catalyzed imino-enamine tautomerism reported above and isolated by HPLC. 

*2-((3-(pyridin-3-yl)-2,5-dihydroisoxazol-5-yl)methyl)benzo[d]isothiazol-3(2H)-one 1,1-dioxide* (**1e**). Colourless oil. ^1^H-NMR (400 MHz, CDCl_3_): *δ* 3.96 (dd, ^2^*J* = 14.8 Hz, ^3^*J* = 7.5 Hz, 1H, CH_2_), 4.10 (dd, ^2^*J* = 14.8 Hz, ^3^*J* = 5.6 Hz, 1H, CH_2_), 4.45 (q, *J* = 7.2 Hz, 1H, H_isoxazoline_), 6.43–6.45 (m, 1H, H_isoxazoline_), 8.66–8.68 (m,1H, Ar), 7.35–7.39 (m, 1H, pyr), 7.63–7.67 (m, 1H, Ar), 7.83–7.85 (m, 1H, Ar), 7.88–7.95 (m, 1H, Ar), 7.98–7.99 (m, 1H, pyr), 8.66–8.68 (m, 1H, pyr), 8.82–8.83 (m, 1H, pyr). Anal. Calcd for C_16_H_13_N_3_O_4_S: C, 55.97; H, 3.82; N, 12.24. Found: C, 55.94; H, 3.84; N, 12.25.

### 3.5. Absolute Configuration Assignment

The assignment of the absolute configuration to the enantiomers of **1i** and **1e** collected at the semipreparative level was determined empirically by circular dichroism correlation, using the enantiomers of an analog of **1i** bearing the phenyl ring instead of the pyridyl ring as references to known stereochemistry [14]. As shown in Figure 5, the enantiomers of the chiral compounds exhibited intense and very similar electronic CD spectra in the ethanol solution [14]. This indicates that the first eluted enantiomers of the imine and enamine forms of **1** had an *R* configuration, and the more retained enantiomers had an *S* configuration.

## 4. Conclusions

In summary, the selectivity of chiral stationary phases containing amylose (3,5-dimethylphenylcarbamate) as a selector made it possible to separate the enantiomers of the imine and enamine forms of **1** in a single-run. The enantio- and tautomer-selective HPLC method was exploited to investigate the influence of various factors (e.g., temperature, type of reaction solvent, concentration of basic catalysts) on the isomeric imine/enamine ratio.

As established by off-column kinetic experiments, the isomeric interconversion of **1** was promoted by a basic catalyst and proceeded without concomitant racemization of the stereocenter C_4_. 

Under the conditions used in the last step of the synthesis of **1** (solvent reaction: ethyl acetate/TEA, temperature: 25 °C, reaction time: 24 h) (Scheme 1), the imine-enamine conversion was blocked, and only the imine form, which was less stable than the enamine form, was present, as demonstrated by the HPLC analysis.

In other words, the reaction proceeded to produce a pair of enantiomers of the kinetically favored imine form.

Conversely, in methanol, in the presence of a basic catalyst, the thermodynamically favored enamine form was mainly obtained. Therefore, the synthesis of isoxazoline derivatives could be modulated favoring the imine or enamine form simply by switching the reaction conditions from a kinetic to a thermodynamic control and vice versa.

Under biomimetic conditions, the tautomeric equilibrium was completely shifted in favor of the imine form, which represented 99% of the isomeric mixture. Since the control of configurational integrity is a critical aspect in the design of chiral biologically active molecules, the kinetic results presented in this study can provide the chemical pathway to rationally design drug candidates based on isoxazoline scaffold with configurational stability compatible with the pharmacological time scale.

## Figures and Tables

**Table 1:** Experimental kinetic constants (k_obs_) and half-life times (t_0.5_) of the pseudo-first-order isomerization imine/enamine process of **1** determined by the off-column HPLC method.

Reaction Solvent	T(°C)	k_(I_→_E)_ (s^−1^)	k_(E_→_I)_ (s^−1^)	t_0.5(I_→_E)_ (min)	t_0.5(E_→_I)_ (min)
Methanol/TEA (0.1%)	20	1.64 × 10^−4^	2.89 × 10^−5^	70	400
Methanol/TEA (0.1%)	25	1.69 × 10^−4^	3.18 × 10^−5^	68	363
Methanol/TEA (0.1%)	30	3.36 × 10^−4^	7.07 × 10^−5^	34	163
Methanol/TEA (0.1%)	35	1.20 × 10^−3^	2.65 × 10^−4^	10	44
Methanol/TEA (0.2%)	20	2.66 × 10^−4^	4.70 × 10^−5^	43	246
Methanol/TEA (0.2%)	25	3.64 × 10^−4^	6.84 × 10^−5^	32	169
Methanol/TEA (0.2%)	30	8.06 × 10^−4^	1.65 × 10^−4^	14	70
Methanol/TEA (0.2%)	35	1.79 × 10^−3^	4.23 × 10^−4^	6	27
Methanol/TEA (0.4%)	20	4.39 × 10^−4^	7.75 × 10^−5^	26	149
Methanol/TEA (0.4%)	25	6.81 × 10^−4^	1.09 × 10^−4^	17	106
Methanol/TEA (0.4%)	30	1.65 × 10^−3^	3.37 × 10^−4^	7	34
Methanol/TEA (0.4%)	35	2.86 × 10^−3^	6.75 × 10^−4^	4	17
Ethanol/TEA (10%)	50	6.1 × 10^−5^	3.0 × 10^−5^	189	391
Ethanol/TEA (10%)	55	8.6 × 10^−5^	4.6 × 10^−5^	135	250
Ethanol/TEA (10%)	60	9.5 × 10^−5^	5.0 × 10^−5^	122	232
Ethanol/TEA (10%)	65	1.3 × 10^−5^	8.2 × 10^−5^	87	141
Ethanol/TEA (4%)	60	6.4 × 10^−5^	3.4 × 10^−5^	179	340
Ethanol/TEA (6%)	60	8.0 × 10^−5^	4.2 × 10^−5^	145	275
Ethanol/TEA (8%)	60	8.8 × 10^−5^	4.6 × 10^−5^	132	250
1-Propanol/TEA (10%)	60	The isomeric ratio remained unchanged within 24 h			
Ethyl acetate/TEA (10%)	60	The isomeric ratio remained unchanged within 24 h			
Acetonitrile/TEA (10%)	60	The isomeric ratio remained unchanged within 24 h			

## Data Availability

Data sharing not applicable.

## Supplementary Materials

The following supporting information can be downloaded at: https://www.mdpi.com/article/10.3390/molecules28186518/s1, Figure S1: UV spectra of **1e** and **1i**; Figure S2: Effect of temperature on the %**1i** at equilibrium.
