# Supplementary figures and images for: Kinetic Study on the Base-Catalyzed Imine-Enamine Tautomerism of a Chiral Biologically Active Isoxazoline Derivative by HPLC on Amylose Tris(3,5-dimethylphenylcarbamate) Chiral Stationary Phase
Source: Molecules. 2023 Sep 8;28(18):6518. doi: 10.3390/molecules28186518 (PMC10538074; doi:10.3390/molecules28186518)

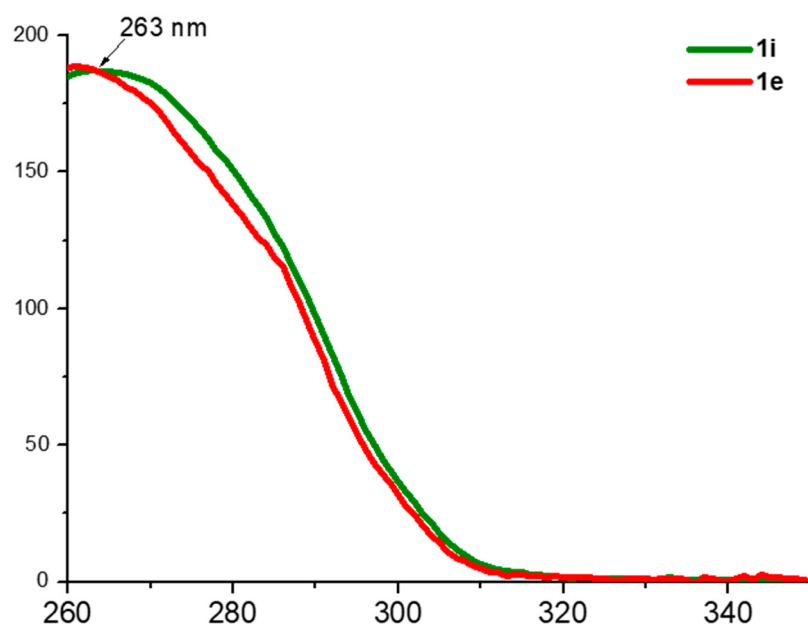

**Figure S1.** UV spectra of **1e** and **1i**.

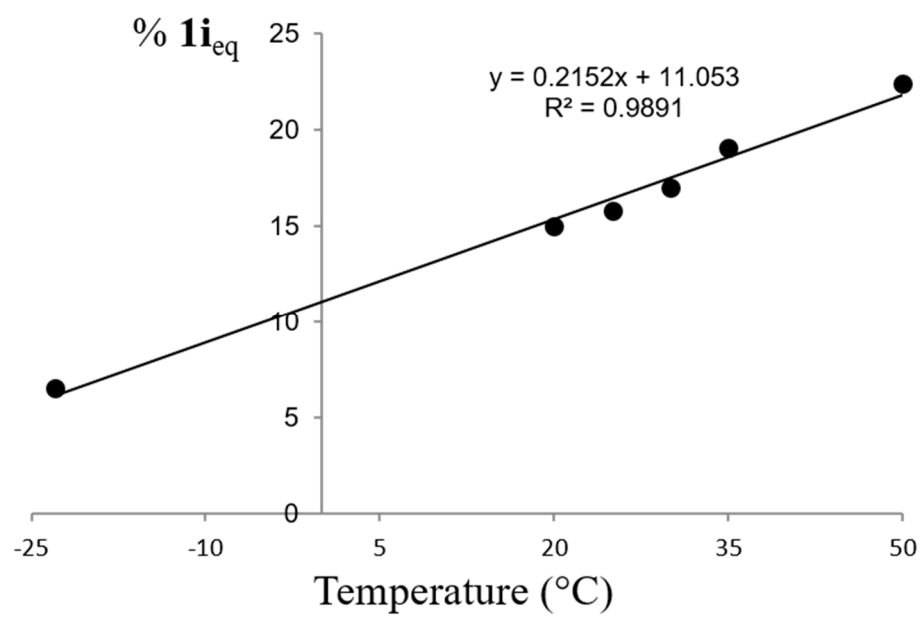

**Figure S2.** Effect of temperature on the %**1i** at equilibrium.

Supplement: Supplementary file 1 [file molecules-28-06518-s001.zip › molecules-2563700-supplementary.pdf]
